# Supplementary material for: Core-like groups result in invalidation of identifying super-spreader by k-shell decomposition
Source: Sci Rep. 2015 May 6;5:9602. doi: 10.1038/srep09602 (PMC5386204; doi:10.1038/srep09602)
Supplement: Supplementary Information — Supporting Information [file srep09602-s1.pdf]

Supporting Information for

Core-like groups result in invalidation of identifying super-spreader by k-shell decomposition

Ying Liu, Ming Tang, Tao Zhou and Younghae Do

Table S1. Proportion of nodes in high shells in the studied real networks. d indicates the shell ranking difference of a shell from the highest shell. $d = 0$ corresponds to the highest shell, $d = 1$ corresponds to the nearest shell from the highest shell. For example, the highest shell of CA-Hep is 31-shell, while the second highest shell is 23-shell. Thus, $d = 0$ corresponds to 31-shell, and $d = 1$ correspond to 23-shell.

**Network**	$d=0$	$d=1$	$d=2$	$d=3$	$d=4$	$d=5$	$d=6$	$d=7$	$d=8$	$d=9$
Router	0.52%	0.80%	1.25%	2.15%	3.25%	6.53%	85.50%	0	0	0
Emailcontact	0.33%	0.05%	0.01%	0.03%	0.02%	0.02%	0.29%	0.10%	0.06%	0.26%
AS	0.31%	0.03%	0.02%	0.03%	0.03%	0.04%	0.03%	0.03%	0.06%	0.06%
Email	1.06%	9.62%	10.33%	9.80%	7.50%	8.83%	11.56%	7.33%	8.83%	11.47%
CA-Hep	0.37%	0.28%	0.24%	0.22%	0.12%	2.07%	3.62%	6.44%	10.23%	13.36%
Hamster	1.25%	4.25%	2.75%	0.70%	2.75%	1.85%	1.30%	6.25%	2.90%	3.40%
PGP	0.38%	0.02%	0.67%	0.06%	0.03%	0.02%	0.19%	0.02%	0.23%	0.01%
Netsci	2.38%	4.22%	5.54%	6.07%	26.91%	24.80%	22.96%	7.12%	0	0
Astro	0.38%	0.38%	0.35%	0.38%	0.33%	0.30%	0.28%	0.01%	0.29%	0.56%

Text S1. Explanation of the k_S imprecision demonstrated in Fig. S1. In Router, the imprecision is under 0.1 at first, and then rises at around $p \approx 0.15$. As the number of nodes in 1-shell accounts for 85.5% of the network size, the rising results from the random selection of nodes in 1-shell. In Emailcontact and AS, within in 20% of the network size, the imprecision is low. In Fig. S1 (d)-(f) the imprecision of k_S decreases with p. In Email, the core-like groups, 11-shell, accounts for 1.1% of the network size. From 10-shell, the spreading efficiency decreases with shells. The number of nodes in 10-shell accounts for about 10% of the network size. This results in the sharp decrease of k_S impression until $p \approx 0.11$. In CA-Hep, the 31-shell, 23-shell, 20-shell and 18-shell accounts for about 1% of network size, which corresponds to the high k_S imprecision for $p < 0.01$ (See Fig. 1 in main text). Next to the 18-shell is the 9-shell. From the 9-shell, the spreading efficiency decreases with shells. The proportion of nodes in shells 9, 8, 7, 6 is 0.1%, 2%, 3.6%, 6.4% respectively. Thus at $p \approx 0.13$, the imprecision of k_S goes to a low value. In Hamster, the innermost 24-shell accounts for 1.25% of the network size, which corresponds to the high k_S imprecision before $p = 0.0125$ (See Fig. 1 in main text). Then the k_S imprecision decreases with p until $p \approx 0.09$, which corresponds to the high spreading efficiency of 22-shell and 21-shell. In PGP, from the 31-shell to 22-shell, accounting for 1.4% of network size, the spreading efficiency decrease from the inner shell to periphery shell, which corresponds to the low k_S imprecision (See Fig. 1 in main text). Then from 21-shell to 11-shell, locally densely connected groups occurs. The proportion of nodes of these shells accounts for 2.1% of the network size. Thus , at $p \approx 0.035$, the k_S imprecision goes down. In Netsci, core nodes accounts for 2.4% of network size, that corresponds to the the low k_S imprecision before $p = 0.024$. Then in shell 7 and 6 , where the proportion of nodes are 4.2%, 5.5% respectively, locally densely connected groups occurs, which corresponds to the highest k_S imprecision at $p \approx 0.12$. The proportion of nodes in 5-shell is 6.1%, and since then, the k_S imprecision begins to decrease. In Astro, the low k_S imprecision before $p = 0.015$ (See Fig. 1 in main text) corresponds to the 51-shell and above. The 48-shell is a local group, accounting for 0.3% of the the network size. This corresponds to the sharp rise at $p \approx 0.015$. Then the k_S imprecision goes down.

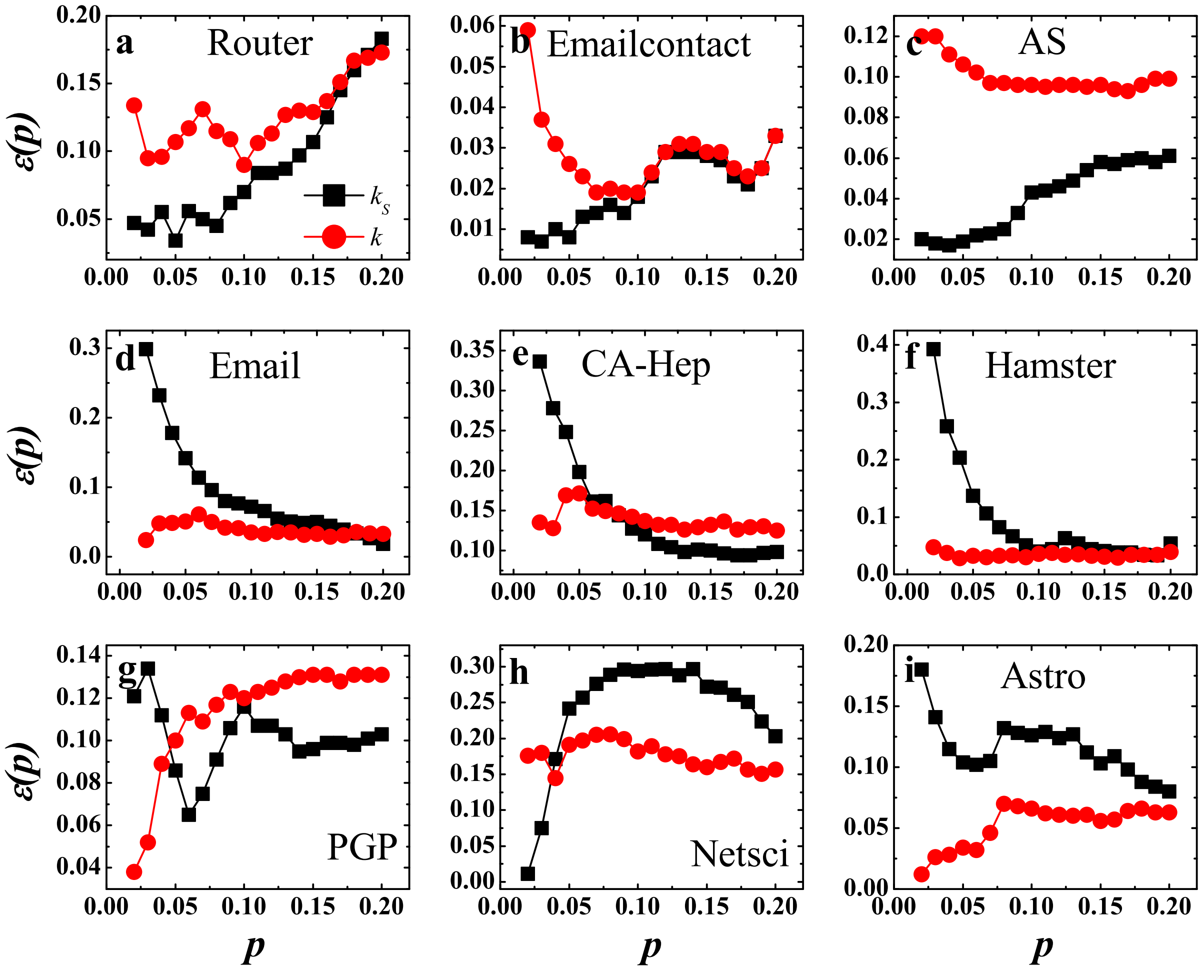

Figure S1. The imprecision of k_S and k as a function of p for nine real networks. p is the proportion of nodes calculated, ranging from 0.02 to 0.2.

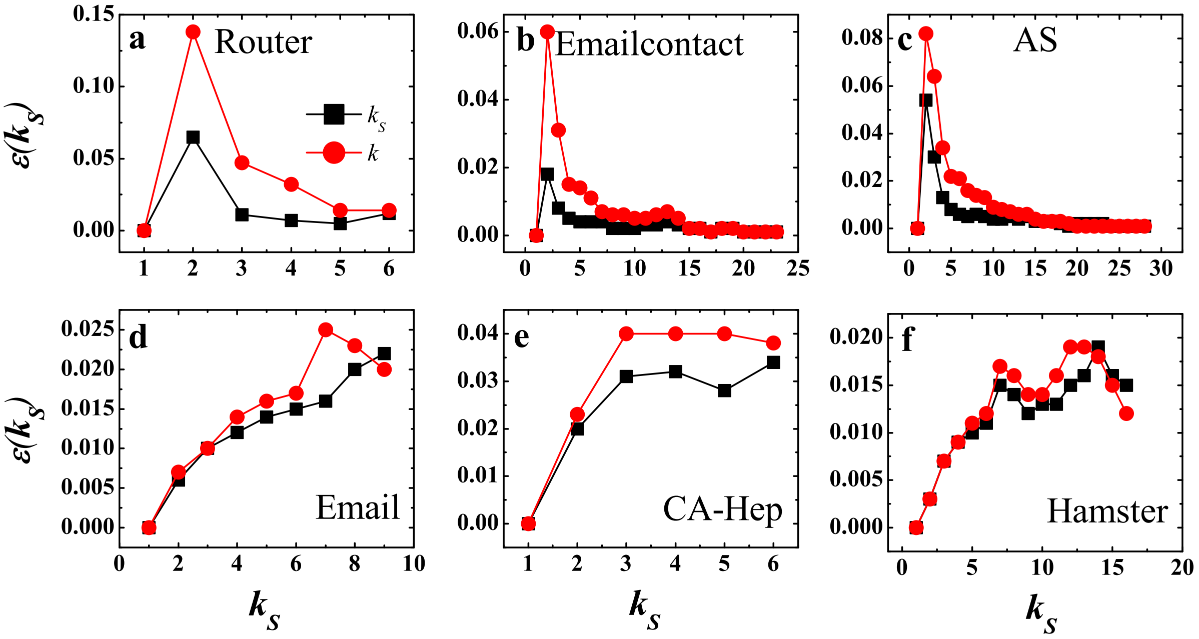

Figure S2. The imprecision of k_S and k as a function of k_S for degree-preserving randomized networks. In all the networks shown in (a)-(f), the k_S imprecision is very low, under the value of 0.07, and in most cases lower than k imprecision. For the randomized networks of Email and Hamster, although the k_S imprecision is slightly higher than that of degree in some shells, the absolute values are very low, under 0.025. This indicates that the k-shell strategy is more effective than or at least as well as degree in most cases.

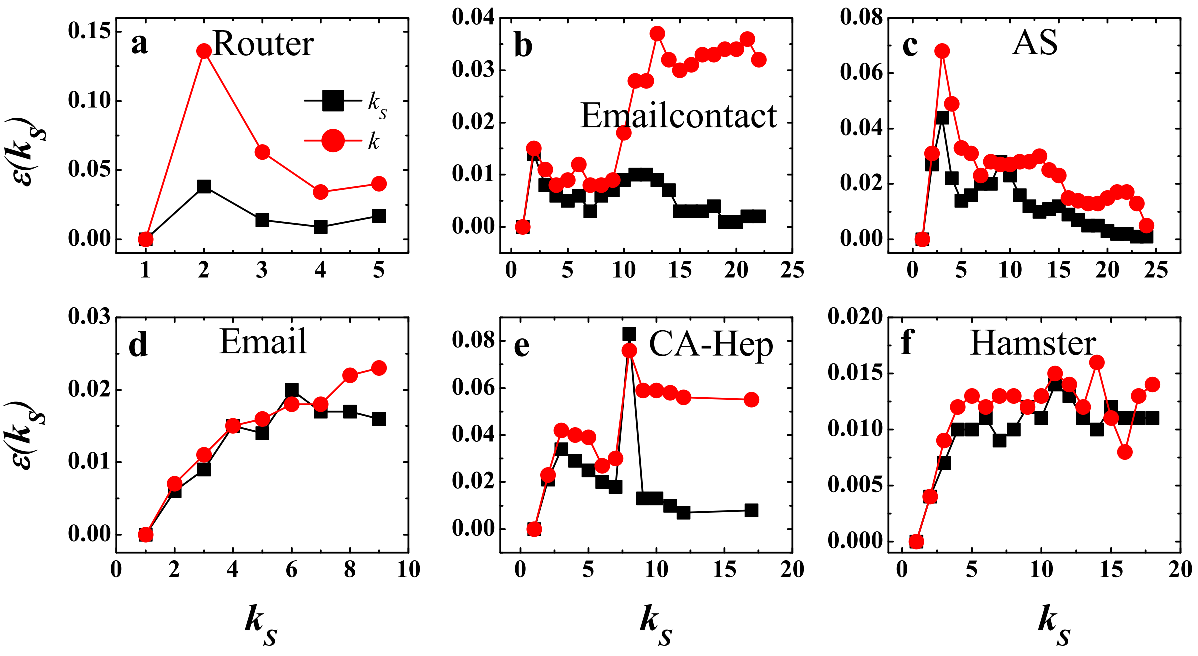

Figure S3. The imprecision of k_S and k as a function of k_S for degree-degree correlation preserving randomized networks. The imprecision of k_S is very low in high shells and is lower than that of k.

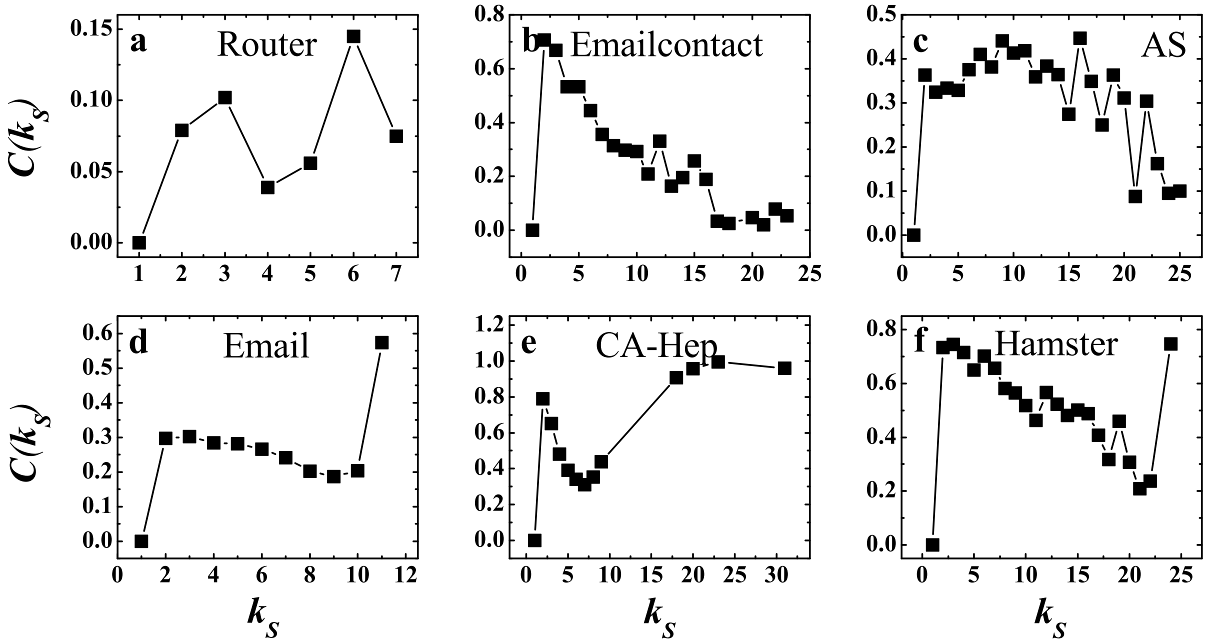

Figure S4. Clustering coefficient of shells for the real networks. (a), (b), (c) The average clustering coefficient is smaller than 0.1 in the innermost core shell. (d), (e), (f) The average clustering coefficient is greater than 0.5 in the innermost core.

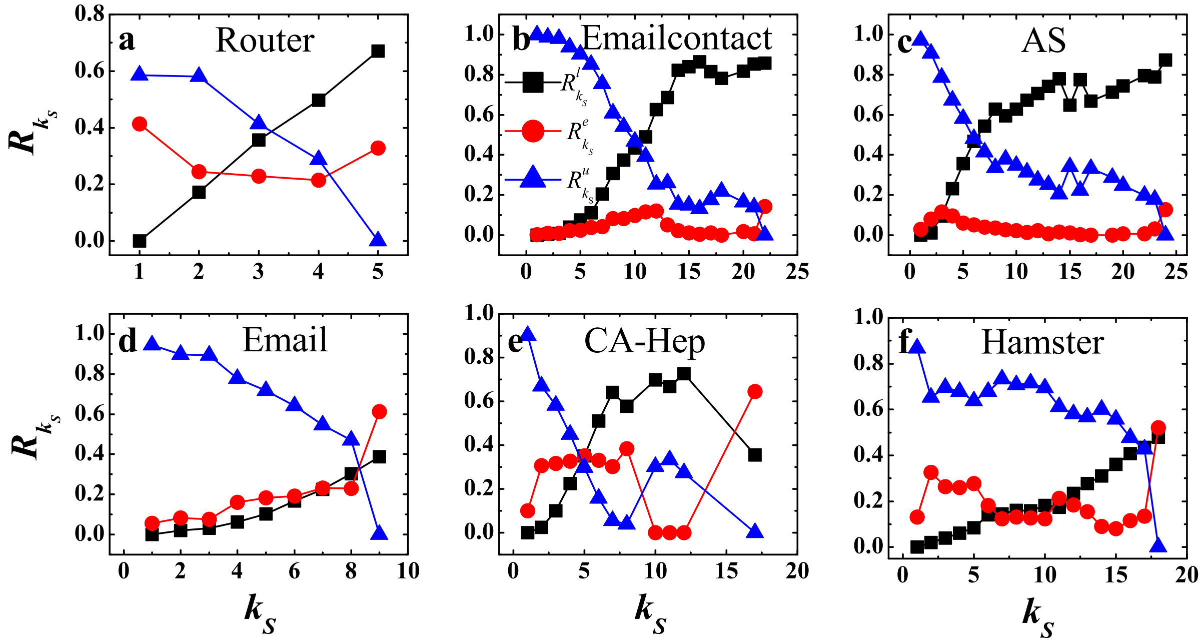

Figure S5. Link strength of shells for degree-degree correlation preserving randomized networks . The link strength of each shell to its lower shells $R^l_{k_S}$ (black squares), equal shell $R^e_{k_S}$ (red circles), and upper shells $R^u_{k_S}$ (blue triangles) in the degree-degree correlation preserving randomized networks are represented. (a), (b), (c) $R^l_{k_S}$ is much larger than $R^e_{k_S}$ in high shells. (d), (e), (f) $R^l_{k_S}$ is strongly promoted and is always larger than $R^e_{k_S}$ in high shells in CA-Hep (e) and Hamster (f), although in Email (d) there is no obvious promotion.

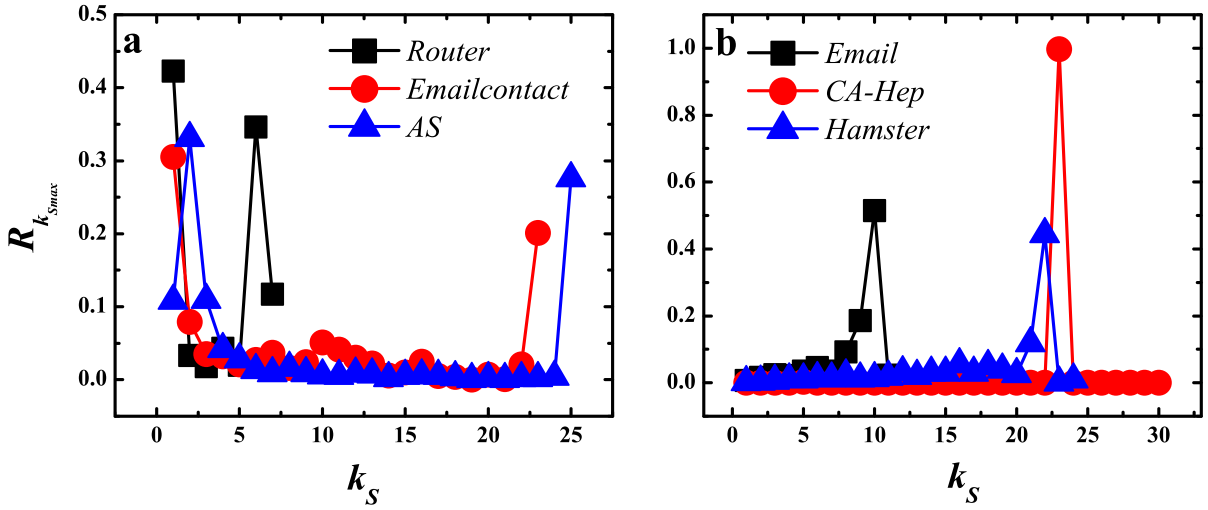

Figure S6. Link strength of the second innermost shell to each shell of the network. (a) Similar to the core, the second innermost shell are well connected to other parts of the network in Router (black squares), Emailcontact (red circles) and AS (blue triangles). (b) The link ratio within the second highest shell is lower than 0.6 in Email (black squares) and Hamster (blue triangles), but is still close to 1.0 CA-Hep (red circles).

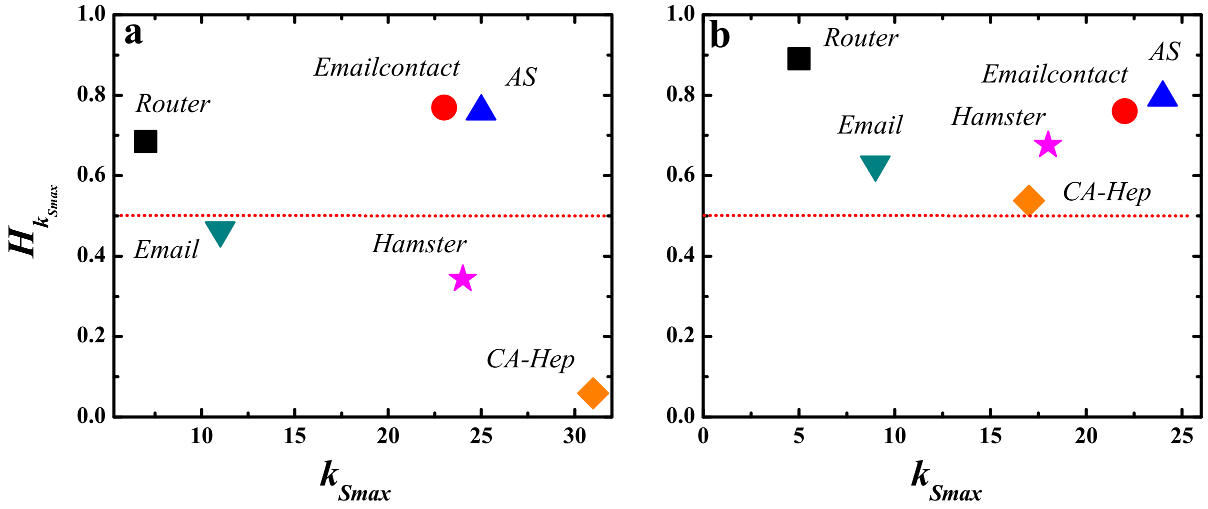

Figure S7. Link entropy of the innermost core for the real networks and their randomized version. (a) Link entropy of the innermost core for the real networks. (b) Link entropy of the innermost core for the degree-degree correlation preserving randomized networks. In all the randomized networks, the core entropy is above 0.5.

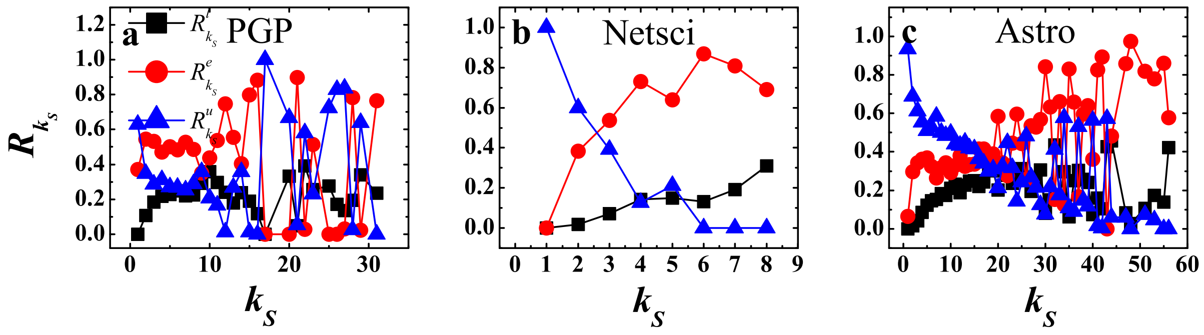

Figure S8. Link strength of shells for three real networks. The link strength of each shell to its lower shells $R^l_{k_S}$ (black squares), equal shell $R^e_{k_S}$ (red circles) and upper shells $R^u_{k_S}$ (blue triangles) are represented. For those core-like groups, 21-shell, 16-shell and 15-shell in PGP, 7-shell and 6-shell in Netsci and 48-shell and 30-shell in Astro, $R^e_{k_S}$ is much higher than $R^l_{k_S}$.

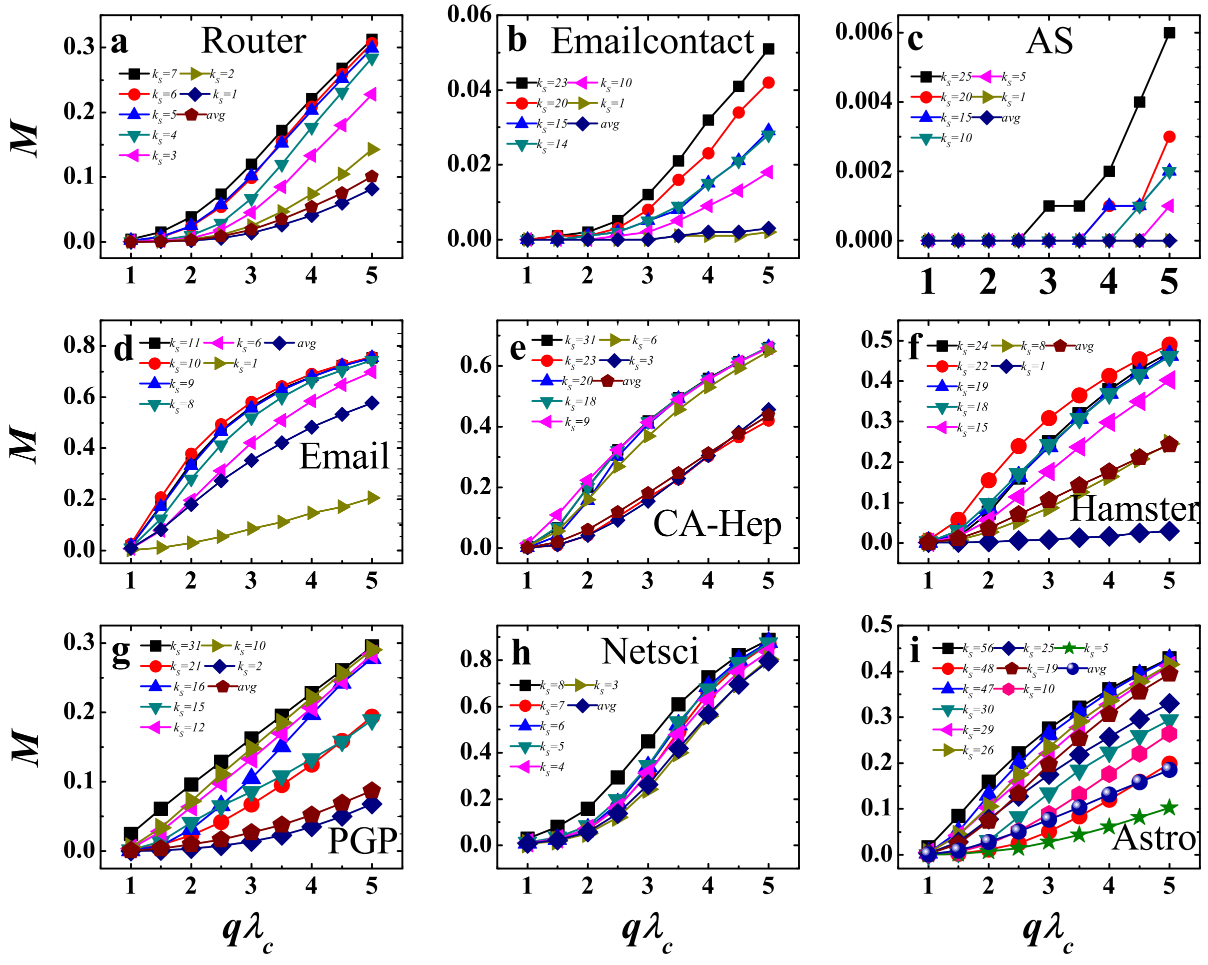

Figure S9. Infected population of k_S shells as a function of infection probability, which is q times of the epidemic threshold λ_c, q ranges from 1 to 5. (a), (b), (c) In the first group, high shells are consistently reaching a higher infection population than low shells. (d), (e), (f) In the second group, the innermost cores have a relatively low efficiency than low shells. (g), (h), (i) In the third group, the innermost cores have the highest spreading efficiency. But there exist some shells of high k_S index that have a lower efficiency than adjacent shells. They are 21-shell, 16-shell and 15-shell in PGP, 7-shell and 6-shell in Netsci and 48-shell and 30-shell in Astro.

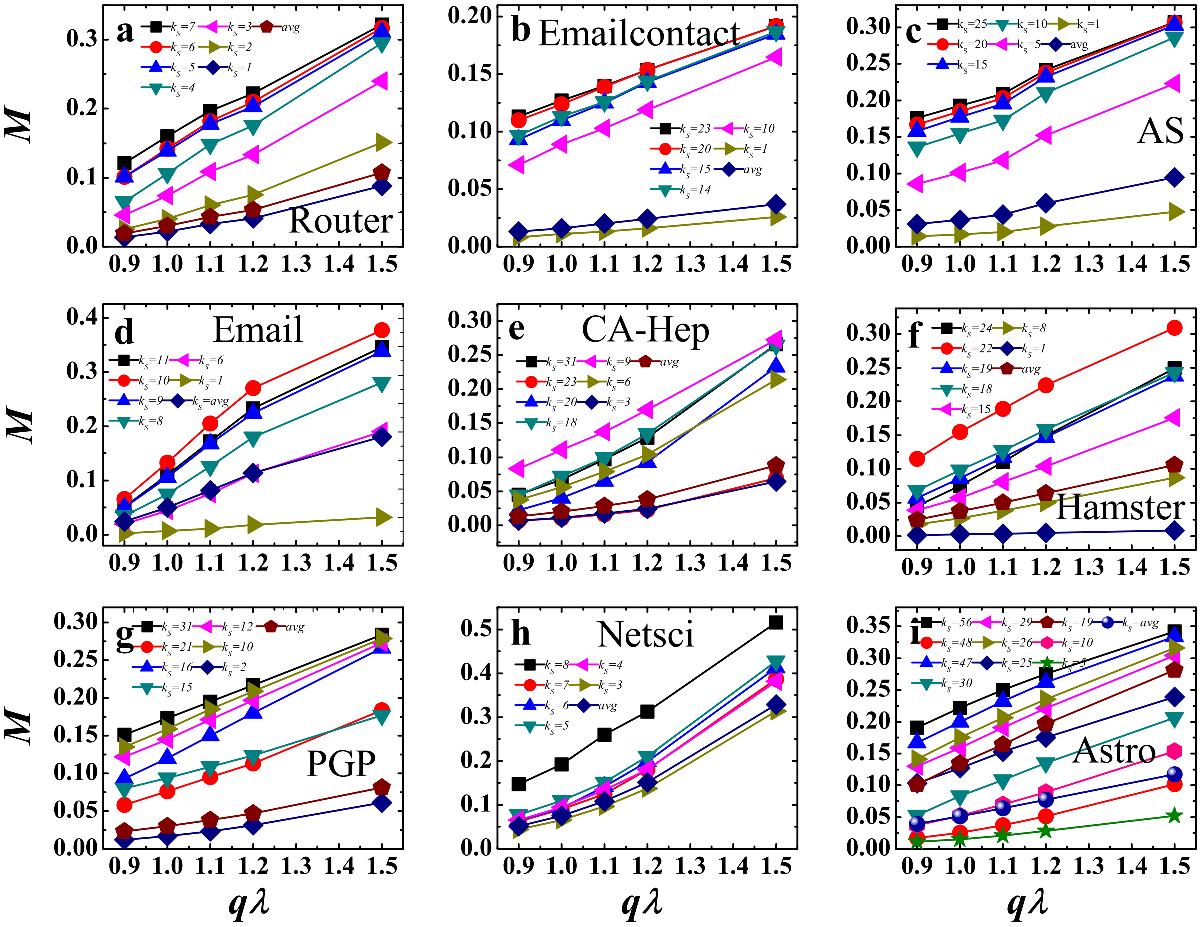

Figure S10. Infected population of k_S shells as a function of infection probability, which is q times of the infected probability λ, q ranges from 0.9 to 1.5. The relative spreading efficiency of shells is the same as the spreading when the infection probability is around λ_c as shown in Figure S9.
